# Telomere length and genetics are independent colorectal tumour risk factors in an evaluation of biomarkers in normal bowel

**DOI:** 10.1038/bjc.2017.486

**Published:** 2018-02-13

**Authors:** Ceres Fernandez-Rozadilla, Christiana Kartsonaki, Connor Woolley, Michael McClellan, Deb Whittington, Gareth Horgan, Simon Leedham, Skirmantas Kriaucionis, James East, Ian Tomlinson

**Affiliations:** 1 Molecular and Population Genetics Laboratory University of Oxford, Oxford OX3 7BN, UK; 2 Clinical Trial Service Unit and Epidemiological Studies Unit, Nuffield Department of Population Health, Old Road Campus, Oxford OX3 7DQ, UK; 3 Ludwig Institute for Cancer Research, University of Oxford, Old Road Campus Research Building, Roosevelt Drive, Oxford OX3 7DQ, UK; 4 Translational Gastroenterology Unit, Experimental Medicine Division, Nuffield Department of Clinical Medicine, John Radcliffe Hospital, Oxford OX3 9DU, UK; 5 Gastrointestinal Stem Cell Biology Laboratory, Oxford Centre for Cancer Gene Research and; 6 Institute of Cancer and Genomic Sciences, College of Medical and Dental Sciences, University of Birmingham, Vincent Drive, Edgbaston, Birmingham B15 2TT, UK

**Keywords:** colorectal cancer, risk, intermediate phenotypes, biomarkers, prevention

## Abstract

**Background:**

Colorectal cancer (CRC) screening might be improved by using a measure of prior risk to modulate screening intensity or the faecal immunochemical test threshold. Intermediate molecular biomarkers could aid risk prediction by capturing both known and unknown risk factors.

**Methods:**

We sampled normal bowel mucosa from the proximal colon, distal colon and rectum of 317 individuals undergoing colonoscopy. We defined cases as having a personal history of colorectal polyp(s)/cancer, and controls as having no history of colorectal neoplasia. Molecular analyses were performed for: telomere length (TL); global methylation; and the expression of genes in molecular pathways associated with colorectal tumourigenesis. We also calculated a polygenic risk score (PRS) based on CRC susceptibility polymorphisms.

**Results:**

Bowel TL was significantly longer in cases than controls, but was not associated with blood TL. PRS was significantly and independently higher in cases. Hypermethylation showed a suggestive association with case:control status. No gene or pathway was differentially expressed between cases and controls. Gene expression often varied considerably between bowel locations.

**Conclusions:**

PRS and bowel TL (but not blood TL) may be clinically-useful predictors of CRC risk. Sample collection to assess these biomarkers is feasible in clinical practice, especially where population screening uses flexible sigmoidoscopy or colonoscopy.

Colorectal carcinoma (CRC) is one of the most common forms of malignancy (Ferlay *et al*, 2013). It is considered a complex disease, with both inherited and environmental factors involved in predisposition (Houlston and Peto, 2004). Genome-wide association studies (GWAS) have so far identified over 30 common polymorphisms (SNPs) associated with CRC and adenoma risk in the general population (Broderick *et al*, 2007; Tomlinson *et al*, 2007, 2008, 2011; Houlston *et al*, 2008, 2010; Tenesa *et al*, 2008; Dunlop *et al*, 2012; Whiffin *et al*, 2014).

Screening for benign polyps and their removal by colonoscopy can reduce CRC risk (Zauber *et al*, 2012). Many European countries now offer population screening programmes, based primarily on faecal occult blood tests (FOBT) (Robertson and Imperiale, 2015). Despite the effectiveness of these programmes, the burden of screening is likely to increase considerably and CRC will continue to be a major killer. It would therefore be useful to improve screening by targeting it to those at highest risk. Individualised risk prediction is generally infeasible outside the Mendelian CRC syndromes (Dunlop *et al*, 2013). It remains possible, however, that risk prediction is still clinically useful when applied to population-level screening because of nthe following reasons: (i) the unknown and/or unmeasurable risks are distributed among large numbers of individuals and (ii) population stratification by prior risk is an improvement on the current screening that is targeted solely by age.

Another potentially useful measure of disease risk is the intermediate molecular phenotype, since this can hypothetically capture both known and unknown factors that act through a common mechanism. For example, there are at least 7 CRC SNPs with putative effects on bone morphogenetic protein (BMP) signalling, and a measure of BMP pathway activity might, therefore, capture the effects on BMP signalling of the following: (i) these SNPs, (ii) other unknown SNPs, (iii) rare variants in the same pathway, and (iv) unknown environmental factors. Other intermediate phenotypes with potential effects on CRC risk include telomere length (TL) (von Zglinicki, 2002) and DNA methylation (Ehrlich, 2002). These phenotypes have usually been assessed in a more convenient sample such as peripheral blood, rather than in the bowel itself. While many biomarkers have been postulated to predict CRC risk (Ishihara *et al* 2008), most of these are not established risk factors and there exist relatively few previous studies of intermediate molecular markers (Alexander *et al*, 2017).

The polygenic risk score (PRS) – a quantitative score of susceptibility caused by common genetic variants (Dudbridge, 2013) – is another measure of CRC risk that may be clinically useful (Machiela *et al*, 2011). The predictive value of the CRC PRS has been assessed in previous studies (Dunlop *et al*, 2013; Frampton *et al*, 2016). However, these have only considered carcinoma risk and none included polyps or performed a prospective assessment.

In this study, we have searched for associations between a set of candidate intermediate molecular markers and case:control status in individuals undergoing colonoscopic screening for colorectal tumours. We have also assessed a PRS based on the major CRC predisposition SNPs. Our results have potential importance and clinical applicability for risk prediction models that can be used to modulate population screening for CRC.

## Materials and methods

### Patients and samples

Colorectal biopsies were obtained from 317 individuals of white UK origin undergoing colonoscopy in Oxford. Indications for colonoscopy included: family history of CRC (*N*=15); symptoms compatible with CRC (*N*=164); follow-up for previous polyps/CRC (*N*=97); FOBT-positive UK National Bowel Cancer Screening patients (*N*=91), and rectal prolapse or diverticulitis (*N*=16). ∼3 mm^3^ biopsies of normal bowel epithelium were taken from up to three sites (rectum, distal colon and proximal colon), together with 10 ml peripheral blood. Personal and family histories of bowel disease and the basic demographic data were recorded. Participants were placed into one of the following three categories: (1) polyp cases—individuals with personal history of adenomatous and/or serrated polyps; (2) CRC cases—individuals with personal history of carcinoma; and (3) controls—individuals with no polyps or CRC in the present or past. Individuals not fulfilling these criteria or with other pathologies, such as inflammatory bowel disease, were excluded (Table 1). Ethical approval was obtained from the Oxfordshire Research Ethics Committee A (REC 10/H0604/72). All patients provided informed consent, according to the tenets of the Declaration of Helsinki.

### DNA/RNA extraction

Each biopsy was submerged in RNAlater (Life Technologies). DNA and RNA were extracted with the AllPrep DNA–RNA Mini kit (Qiagen, Manchester, UK). Where multiple biopsies had been taken from the same patient, each individual biopsy was analysed, except that for assays in which a single DNA-based measure per patient was required, where an aliquot of DNA from each available location was pooled in equal proportions. For RNA samples, an aliquot was DNase-treated and reverse transcribed into cDNA. We estimated the typical ratio of epithelium–to–mesenchyme in biopsies to be 1:1.

### Two-stage design

Participants were recruited in two stages: Phase 1 (54 cases and 47 controls was collected between November 2012 and July 2013 based on the indications described above for colonoscopy; replication Phase 2, comprising 157 cases and 59 controls, was collected between July 2013 and March 2014 based on the same criteria. Phase 1 was analysed for all DNA and RNA markers (Supplementary Methods). In Phase 2, all patients were analysed for all the DNA-based markers, and a subset of 103 cases and all controls were analysed for the expression of genes that had evidence in Phase 1 of an association with case:control status at a nominal *P*=0.05.

### Telomere length assays

We assessed telomere length (TL) in blood and bowel DNA in 262 patients using a modification of Cawthon´'s method (Cawthon, 2009; Jones *et al*, 2012). DNA samples were run in duplicate on a CFX96 system (Bio-Rad, Watford, UK). A curve was established relating the quantity of a standard DNA to ΔCt for the telomere repeat and a single-copy gene (gamma haemoglobin). This provided the expected Ct value (ECt) for the telomere repeat. Corrected Ct (CorrDCt) was calculated by subtracting ECt from observed (OCt), providing a relative measure of telomere length. CorrDCt was always >0 and the higher the CorrDCt, the longer the telomere.

### Genomic DNA methylation assay

Global DNA methylation analyses were performed using reverse-HPLC (Quinlivan and Gregory, 2008) by measuring total 5-methylcytosine (5mC) content in 136 patients. Briefly, 2500 ng genomic DNA were RNase-treated in NEB2.1 buffer and incubated for 1 h at 37 C, purified with ethanol and lyophilised. The sample was re-suspended in 50 μl RNase-free water and hydrolysed overnight in a solution containing 45 mM NaCl, 9 mM MgCl_2_, 9 mM Tris pH 7.9, ⩾250 U/ ml^−1^ Benzonase (Sigma), 50 mU/ ml^−1^ Phosphodiesterase I, ⩾20 U/ ml^−1^ Alkaline phosphatase, 46.8 ng/ ml^−1^ EHNA hydrochloride, 8.64 μM deferoxamine. Protein components were removed with Amicon centrifugal filter units (3 kDa cutoff, Millipore, Watford, UK) and resolved with an Agilent UHPLC 1290 (Stockport, UK) instrument fitted with Eclipse Plus C18 RRHD 1.8 μm, 2.1 × 150 mm column. Buffer A was 100 mM ammonium acetate, pH 6.5; buffer B was 40% acetonitrile, and the flow rate 0.4 ml min^−1^. The gradient was between 1.8 and 100 of 40% acetonitrile. Methylation was also assayed in blood and bowel DNA separately.

### Molecular pathway activity assessment

Using real-time, quantitative PCR (qPCR), eight molecular pathways were investigated, which were either related to CRC development or reflecting cellular processes linked to tumourigenesis (Supplementary Table 1). To assess these, we identified 33 genes as pathway end points. Gene expression assays were conducted on an ABI900HT real-time instrument (Applied Biosystems, Foster City, CA, USA), using TaqMan (ThermoScientific, Waltham, MA, USA) molecular probes. As per the recommendations in the current qPCR guidelines, we optimised the normalisation procedure by choosing the best-suited set of housekeeping genes. Given that a literature search did not yield did not yield any unambiguous results, we tested 11 endogenous genes (Supplementary Table 1). The optimal set for normalisation was identified using qBase+. Normalisation was then carried out, using a ΔΔCt method for relative quantification.

### SNP genotyping and polygenic risk scores

KASPar genotyping technology (LCGenomics) was used to generate a 25 SNP genotype profile of each of 310 patients (Supplementary Table 2). PRSs were calculated for the 293 patients with ⩾22 successfully genotyped SNPs according to:


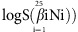


where *β* is the Ln(odds ratio) for each risk allele and N is the number of risk alleles. The missing data points were substituted by the population mean.

### Statistical analyses

These were performed with Stata-v.11 (StataCorp LP, TX, USA) and R (http://www.r-project.org/). For gene expression analysis, individual genes were assessed for associations with case:control status using linear regression fitted with generalised estimated equations (GEE). The activity of each pathway was then generated by carrying out principal component analysis on all gene expression variables for that pathway, and taking the first principal component as a summary pathway value. For DNA phenotypes (TL and methylation), logistic regression was fitted, adjusting by age, sex, phase and experimental batch, where appropriate. Where possible, differences between cases and controls were also evaluated separately for each of the three locations in the large bowel. False discovery rate (FDR) corrections were performed using the Benjamini & Hochberg method (Benjamini and Hochberg, 1995) and significance was assumed at FDR-corrected *q*=0.05. Results are reported below as a meta-analysis of the two Phases, unless otherwise specified. Given the absence of evidence of heterogeneity between Phases, all variables associated with case:control status at nominal *P*=0.05 were then included in a reverse stepwise multivariable logistic regression analysis, and variables associated with case:control status at *P*=0.05 were retained in the final model. Case-only analyses used multivariate logistic regression models including age, sex and phase.

## Results

### Telomere length

**Figure 1:**
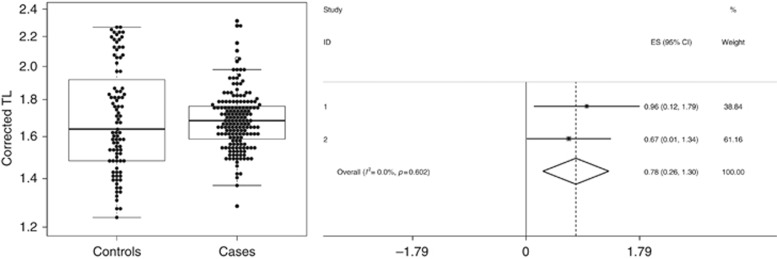
**Association of colorectal telomere length with case:control status.** (**A**) Beeswarm plot of CorrDCt TL values corrected for age in cases and controls with overlying boxplot; (**B**) Forest plot for meta-analysis of TL. CI=confidence interval; TL=telomere length.

After conditioning on age, which co-varied with telomere length as expected, there was an association between longer TLs in the bowel and case status (*P*_meta_=0.003; OR=2.18; 95% CI=1.30–3.67; OR_Q1*vs*Q4_=1.92; 95% CI=_Q1*vs*Q4_=0.87–4.26). Effects in Phases 1 and 2 were consistent (Figure 1). We found no significant difference in bowel TL between the following patients: (i) with polyps at the recruitment colonoscopy or previously (*P*=0.590), (ii) with and without a history of CRC (*P*=0.284), (iii) with polyps or CRC (*P*=0.211), and (iv) with present or previous CRC (*P*=0.664). TL was also independent of family history of colorectal neoplasia (*P*=0.201). Within-patient analysis showed no significant variation in bowel TL by location (Supplementary Figure 1), and this remained true when comparing within-patient TL in biopsies from locations close to the polyp/CRC, compared with those further away (*P*=0.436 for proximal colon, *P*=0.591 for distal colon, *P*=0.055 for rectum).

Of note, blood TL was associated with neither case:control status (*P*=0.989, OR=1.00, 95% CI=0.88–1.14) nor bowel TL (*P*=0.195, OR=1.06 95% CI=0.97–1.15; Supplementary Figure 2), although both bowel and blood TL were negatively correlated with age (*P*=0.000, *r*^2^=−0.03; and *P*=0.000, *r*^2^=−0.04, respectively).

### Methylation

Global methylation (5 mC) levels, as assessed from pooled DNA derived from all three colorectal regions, increased with age as expected (*P*=4 × 10^−5^; OR=1.09; 95% CI=1.05–1.14). We observed a suggestive, but non-significant, increase in global methylation levels in cases compared with controls (*P*_meta_=0.070; OR=1.58; 95% CI=0.96–2.64; Supplementary Figure 3). Blood 5 mC was assayed in a subset of 31 samples, but there was no good evidence of a correlation with bowel 5 mC (*P*=0.139; OR=0.94; 95% CI=0.86–1.02; *r*^2^=−0.27; Supplementary Figure 4). As for TL, we did not find any significant differences between patients with present or past polyps (*P*=0.44), with or without family history (*P*=0.117), polyps or CRC (*P*=0.259) or present *vs* previous CRC (*P*=0.117), and methylation levels were independent of family history (*P*=0.304).

Analyses by location showed significantly lower methylation in the proximal colon compared to the distal colon or the rectum (Supplementary Figure 5). Analyses using only the location-specific measurements of methylation did not yield any significant associations (data not shown).

### Gene expression analysis

We assayed 273 samples from 100 patients for 33 selected genes from 8 pathways (Supplementary Table 1). There was an effect of location on gene expression, with 18 out of 33 genes differentially expressed (at *q*⩽0.05) between the proximal and distal colon, 21 out of 33 between the proximal colon and rectum, and 16 out of 33 between the distal colon and rectum (Supplementary Figure 6); some genes showed monotonically decreased (*ID1*, *ID3*, *OLFM4, PLK1*) or increased (*AXIN2*, *CCND1*, *CDK6*, *E2F1*, *EPHB2*, *ID2*, *KCNH2*, *KCNMA1*, *LRIG1*, *MCM2*, *PCBP4*, *SCNN1D*, *SCNN1G*, *TFDP1*) levels along the proximal colon-distal colon-rectum axis (Supplementary Figure 7). Correlations between the expression of gene pairs are depicted in Supplementary Figure 8. Case:control status was not significantly associated with the expression of any individual gene at a nominal *P*⩽0.05 (Supplementary Table 3). Pathway analysis revealed nominally higher expression of colonocyte differentiation markers in cases (*P*=0.010, *q*=0.080; Supplementary Table 4).

We then examined bowel location-specific gene expression in cases who had one or more tumours in that location *vs* controls with no colorectal tumour anywhere. Cases with distal colon tumour(s) had significantly higher *SCNN1A* expression in that location than controls (*P*=0.004; *q*=0.032; OR=4.09; 95% CI=1.56–10.76, Supplementary Figure 9). This gene showed no evidence of association with status in the other locations and no additional significant associations were found for other genes. With regards to pathway expression by location, colonocyte differentiation and Wnt levels were higher in cases in the proximal colon (*q*=0.040 for both). Stem cell markers and ion channels showed varying expression levels depending on the bowel segment (Supplementary Table 4).

### Assessment of SNP genotypes, bowel TL and status

In logistic regression analysis conditioning on age, sex and family history, PRS was significantly higher in cases than controls (*P*=0.031; OR=51.1; 95% CI=1.45–1803; per quartile OR=1.22; Supplementary Figure 10). PRS was not significantly correlated with bowel TL, methylation or, interestingly, family history (*P*=0.134, *P*=0.114 and *P*=0.640, respectively, full data not shown).

Two of the three telomerase SNPs were significantly associated with bowel TL (for rs10936599, *P*=0.161; for rs2736100, *P*=0.009; for rs2735940, *P*=0.001; non-parametric trend test; Supplementary Figure 11) and also independently with case:control status. No other SNP was associated with case:control status (details not shown).

In reverse stepwise logistic regression analysis, PRS (*P*=0.046; OR=63.5; 95% CI=1.08–3740) and bowel TL (*P*=0.026; OR=1.74; 95% CI=1.07–2.82) were significantly and independently of greater magnitude in cases than controls.

## Discussion

In this study, we examined whether intermediate molecular phenotypes derived from the bowel mucosa were associated with case:control status in a series of individuals undergoing colonoscopic screening for colorectal neoplasia. Such molecular markers potentially predict CRC risk and could be used to modulate population screening. Our principal finding was that cases with colorectal polyps or CRC had longer bowel telomeres, independent of other risk factors. There is a controversy about the effects of telomere length on the risk of many cancers, including CRC (Quin *et al*, 2014; Seguí *et al*, 2014), but almost all studies have measured telomere length in peripheral blood. Our finding of no correlation between telomere length in the colorectum and blood may help to explain the inconsistent results of previous studies. Despite the assay’s speed and simplicity, telomere measurements from the mixed population of cells in peripheral blood may be inappropriate for predicting cancer risk.

SNPs rs2736100 and rs2735940 near *TERT* and rs10936599 near *TERC*^f^ have been associated with CRC risk (Houlston *et al*, 2010; Kinnersley *et al*, 2012; Yang *et al* 2015), but associations with normal bowel TL length have not been explored. rs2735940 and rs2736100 were significantly associated with bowel TL and each was also independently associated with case:control status, consistent with previous reports (Hofer *et al*, 2012).

It has been posited that hypermethylation, resulting from ageing or environmental causes, is a cause of increased cancer risk (Ehrlich, 2002). However, most studies have investigated methylation levels in circulating blood cells. In our study, we found borderline significant differences in global methylation levels between cases and controls. A true association might have been missed due to the fact that our global methylation measure was too general and did not sufficiently capture finer differential methylation patterns in CpG islands and other regions. In addition, the proximal colon had significantly less methylation than the distal colon and rectum, potentially influencing tumourigenic pathways in the different regions of the large bowel (Bufill, 1990).

Gene expression analysis throughout the colorectum detected no significant associations with case:control status for individual genes, and only a nominal association for molecular pathways in the case of colonocyte differentiation markers. Given the non-uniform gene expression patterns observed throughout the bowel for many of the genes analysed (LaPointe *et al*, 2008), we performed association analyses in each location independently and found that expression of *SCNN1A* in the distal colon was different between cases and controls. *SCNN1A* is a potential marker of differentiation and encodes a membrane calcium channel that plays an essential role in maintaining the osmotic gradient in the intestinal epithelium. We were a little surprised, given the importance of variation in the BMP pathway for CRC risk, that we found no evidence of differential activity in this pathway between cases and controls. Many explanations are possible, including limited assay sensitivity, sub-optimal choice of target genes, and spatial and temporal heterogeneity in gene expression.

Our study’s conclusions should be interpreted in the light of limitations and potential confounding factors. While the association between status and bowel TL could have been caused by the presence of tumour(s) rather than *vice versa*, we found no evidence of this, as there were no significant differences between TL in cases with incident CRC or polyps and previous tumours. Moreover, it seems unlikely that polyps could have major effects on sampled normal mucosa that can be more than a metre away in the large bowel, and indeed we did not observe significant intra-patient differences when comparing TL in biopsies close to and distant from a tumour. Second, the fact that both gene expression and global methylation vary throughout the large bowel suggests that location-specific risk models may be more powerful. Our exploratory analyses (details not shown) have demonstrated that because patients frequently have polyp(s) in only one bowel location, location-specific risk models are required with larger sample sets in order to provide adequate statistical power. Third, we caution that, although we deliberately used simple biopsies without the separation of epithelium and mesenchyme, because these represent the sample type most likely to be available in a clinical setting, our measurements were derived from a mixed cellular population, potentially affecting all assays except the PRS. Fourth, the performance of the PRS is likely to be improved in the future by taking into account recently discovered predisposition loci (Zeng *et al*, 2016, amongst others) and polymorphisms specific to the risks of adenomas, serrated polyps and CRC.

In summary, we have shown the potential value of using intermediate molecular markers, and the independent value of the PRS, for predicting colorectal tumour risk. Our data also show the virtues of using biomarkers derived from the colorectum rather than blood. Bowel cancer screening programmes are increasingly using colonoscopy or flexible sigmoidoscopy as a primary modality and rectal biopsies could be obtained safely from participants at limited cost and subjected to molecular analyses that could then be incorporated into algorithms for subsequent screening. While additional studies are required, we suggest that stratification by prior risk could offer a way to improve the cost-effectiveness of population-level large bowel cancer screening populations, whether by prioritising patientsat highest risk or modulating thresholds for further investigation after screening by FIT.

## Figures and Tables

**Table 1:** Numbers of patients included in the study by case

		***N***	**Male/female**	**Mean age (SD), range**	**Clinical phenotype (*****N***)
Phase 1	Controls	47	24/23	54 (17.3), 22–80	
	Cases	54	33/21	67 (8.2), 42–84	A (46), CRC (8)
Phase 2	Controls	59	27/32	52 (16.4), 21–83	
	Cases	157	79/78	65 (9.4), 30–89	A (79), S (37), CRC (41)

Abbreviations: A=adenoma; CRC=colorectal carcinoma; S=serrated polyp; SD=standard deviation.

Numbers of patients included in the study by case:control status, sex, age, and tumour histology.
