# Correction: Telomere length and genetics are independent colorectal tumour risk factors in an evaluation of biomarkers in normal bowel

**DOI:** 10.1038/s41416-018-0111-0

**Published:** 2018-05-21

**Authors:** Ceres Fernandez-Rozadilla, Christiana Kartsonaki, Connor Woolley, Michael McClellan, Deb Whittington, Gareth Horgan, Simon Leedham, Skirmantas Kriaucionis, James E. East, Ian Tomlinson

**Affiliations:** 1 Molecular and Population Genetics Laboratory University of Oxford, Oxford, OX3 7BN UK; 2 Clinical Trial Service Unit and Epidemiological Studies Unit, Nuffield Department of Population Health, Old Road Campus, Oxford, OX3 7DQ UK; 3 Ludwig Institute for Cancer Research, University of Oxford, Old Road Campus Research Building, Roosevelt Drive, Oxford, OX3 7DQ UK; 4 Translational Gastroenterology Unit, Experimental Medicine Division, Nuffield Department of Clinical Medicine, John Radcliffe Hospital, Oxford, OX3 9DU UK; 5 Gastrointestinal Stem Cell Biology Laboratory, Oxford Centre for Cancer Gene Research, Oxford, UK; 6 Oxford NIHR Biomedical Research Centre, University of Oxford, Oxford, UK; 7 Institute of Cancer and Genomic Sciences, College of Medical and Dental Sciences, University of Birmingham, Vincent Drive, Edgbaston, Birmingham, B15 2TT UK

**Correction to:** *Br J Cancer* (2018) **118**: 727–732; 10.1038/bjc.2017.486; published online 13 February 2018

Since the publication of this paper, the authors noticed that James E. East was assigned to the incorrect affiliation. The affiliation information is provided correctly, above.

Furthermore, the authors would like to add the following information to the Acknowledgements section:

