# Supplementary material for: Telomere length and genetics are independent colorectal tumour risk factors in an evaluation of biomarkers in normal bowel
Source: Br J Cancer. 2018 Feb 13;118(5):727–32. doi: 10.1038/bjc.2017.486 (PMC5846076; doi:10.1038/bjc.2017.486)
Supplement: Supplementary Material [file bjc2017486x1.docx]

**SUPPLEMENTARY INFORMATION**

**Supplementary Table 1. Genes analysed for mRNA expression.** Gene IDs, the pathways to which they belong and Taqman assay IDs ([https://www.thermofisher.com/es/es/home/life-science/pcr/real-time-pcr/real-time-pcr-assays/taqman-gene-expression.html#](https://www.thermofisher.com/es/es/home/life-science/pcr/real-time-pcr/real-time-pcr-assays/taqman-gene-expression.html)) are shown. The 11 housekeeping genes (Endogenous control) used for normalisation are also shown.

| **Gene (HGNC code)** | **Pathway or Phenotype** | **Assay ID** |
| --- | --- | --- |
| ID1 | BMP pathway | Hs03676575_s1 |
| ID2 | BMP pathway | Hs04187239_m1 |
| ID3 | BMP pathway | Hs00954037_g1 |
| PMEPA1 | Colonocyte differentiation | Hs00375306_m1 |
| PRKCA | Colonocyte differentiation | Hs00925193_m1 |
| KCNH2 | Colonocyte differentiation | Hs04234270_g1 |
| KCNMA1 | Colonocyte differentiation | Hs01119498_m1 |
| SCNN1A | Colonocyte differentiation | Hs01013028_m1 |
| SCNN1B | Colonocyte differentiation | Hs01548617_m1 |
| SCNN1D | Colonocyte differentiation | Hs00936285_g1 |
| SCNN1G | Colonocyte differentiation | Hs00168918_m1 |
| CDH1 | EMT | Hs01023894_m1 |
| SNAI1 | EMT | Hs00195591_m1 |
| SNAI2 | EMT | Hs00950344_m1 |
| AURKA | Proliferation | Hs01582072_m1 |
| E2F1 | Proliferation | Hs00153451_m1 |
| FOXM1 | Proliferation | Hs01073586_m1 |
| MCM2 | Proliferation | Hs01091564_m1 |
| MKI67 | Proliferation | Hs01032443_m1 |
| PLK1 | Proliferation | Hs00153444_m1 |
| CDK2 | Senescence (p21) | Hs01548894_m1 |
| CDK4 | Senescence (p21) | Hs01565683_g1 |
| CDK6 | Senescence (p21) | Hs01026371_m1 |
| CDKN1A | Senescence (p21) | Hs00355782_m1 |
| PCBP4 | Senescence (p21) | Hs00924118_g1 |
| TFDP1 | Senescence (p21) | Hs00955488_g1 |
| TFDP2 | Senescence (p21) | Hs00963605_m1 |
| LGR5 | Stem cells | Hs00173664_m1 |
| LRIG1 | Stem cells | Hs00394267_m1 |
| OLFM4 | Stem cells | Hs00197437_m1 |
| AXIN2 | Wnt | Hs00610344_m1 |
| CCND1 | Wnt | Hs00765553_m1 |
| EPHB2 | Wnt | Hs00362096_m1 |
| 18S | Endogenous control | Hs99999901_s1 |
| ACTB | Endogenous control | Hs99999903_m1 |
| B2M | Endogenous control | Hs99999907_m1 |
| GAPDH | Endogenous control | Hs99999905_m1 |
| GUSB | Endogenous control | Hs99999908_m1 |
| HPRT | Endogenous control | Hs99999909_m1 |
| PGK1 | Endogenous control | Hs99999906_m1 |
| PPIA | Endogenous control | Hs99999904_m1 |
| RPLP0 | Endogenous control | Hs99999902_m1 |
| TBP | Endogenous control | Hs99999910_m1 |
| TRFC | Endogenous control | Hs99999911_m1 |

**Supplementary Table 2. SNPs used in the PRS analysis.** Data include: locus ID; risk allele, and its frequency (as in the 1000Genomes GBR population); β=ln(Odds Ratio) used in PRS calculation; nearby potential target genes; and the bibliographic reference. These CRC risk polymorphisms were generally discovered in early GWAS of European populations and are therefore likely to have the largest effect sizes of the larger set of about CRC SNPs that now exists.

| **SNP** | **Chr. band** | **Risk allele** | **Risk allele freq. in UK** | **β** | **Nearby gene(s)** | **REFERENCE** |
| --- | --- | --- | --- | --- | --- | --- |
| rs10911251 | 1q25.3 | A | 0.55 | 0.086 | LAMC1 | Whiffin 2014[11] |
| rs6691170 | 1q41 | A | 0.36 | 0.058 | DUSP10 | Houlston 2010[8] |
| rs10936599 | 3q26.2 | G | 0.74 | 0.039 | MYNN, TERC | Houlston 2010[8] |
| rs2736100 | 5p15.33 | T | 0.58 | 0.074 | TERT | Kinnersley 2012[12] |
| rs2735940 | 5p15.33 | G | 0.50 | 0.058 | TERT | Yang 2015[13] |
| rs1321311 | 6p21 | A | 0.20 | 0.095 | CDKN1A | Dunlop 2012[10] |
| rs16892766 | 8q23.3 | C | 0.09 | 0.239 | EIF3H | Tomlinson 2008[5] |
| rs6983267 | 8q24 | C | 0.54 | 0.215 | MYC | Tomlinson 2007[3] |
| rs10795668 | 10p14 | C | 0.62 | 0.113 | Intergenic | Tomlinson 2008[5] |
| rs1035209 | 10q24.2 | T | 0.21 | 0.122 | ABCC2, MRP2 | Whiffin 2014[11] |
| rs3824999 | 11q13.4 | A | 0.49 | 0.077 | POLD3 | Dunlop 2012[10] |
| rs3802842 | 11q23 | G | 0.32 | 0.104 | Intergenic | Tenesa 2008[6] |
| rs3217810 | 12p13.32 | T | 0.11 | 0.174 | CCND2 | Whiffin 2014[11] |
| rs11169552 | 12q13.3 | G | 0.74 | 0.086 | DIP2B, ATF1 | Houlston 2010[8] |
| rs4444235 | 14q22.2 | C | 0.49 | 0.104 | BMP4 | Houlston 2008[7] |
| rs1957636 | 14q22.2 | T | 0.47 | 0.077 | BMP4 | Tomlinson 2011[10] |
| rs16969681 | 15q13.3 | T | 0.04 | 0.166 | GREM1 | Tomlinson 2011[9] |
| rs11632715 | 15q13.3 | A | 0.47 | 0.113 | GREM1 | Tomlinson 2011[9] |
| rs9929218 | 16q22.1 | G | 0.68 | 0.095 | CDH1 | Houlston 2008[7] |
| rs4939827 | 18q21 | T | 0.52 | 0.166 | SMAD7 | Broderick 2007[4] |
| rs10411210 | 19q13.1 | C | 0.87 | 0.140 | RHPN2 | Houlston 2008[7] |
| rs961253 | 20p12.3 | A | 0.37 | 0.113 | Intergenic | Houlston 2008[7] |
| rs4813802 | 20p12.3 | G | 0.38 | 0.086 | BMP2 | Tomlinson 2011[9] |
| rs4925386 | 20q13.33 | C | 0.67 | 0.077 | LAMA5 | Houlston 2010[8] |
| rs5934683 | Xp22.2 | T | 0.31 | 0.068 | SHROOM2 | Dunlop 2012[10] |

**Supplementary Table 3. (a) Gene expression association with case/control status (entire colorectum).** Results are derived from the GEE analysis with expression as the dependent variable, and age, sex and status as the independent variables. We show the robust p-values, effect sizes (β) and standard errors (SE) for Phases separately and combined. P trend is the p value derived from a trend test on differential expression depending on location.

| Gene | p trend |  | p phase1 |  | β phase1 | SE phase1 | p phase2 | β phase2 | SE phase2 | p phase1+2 | β phase1+2 | SE phase1+2 |
| --- | --- | --- | --- | --- | --- | --- | --- | --- | --- | --- | --- | --- |
| AURKA | 0.08  0.00  0.00  0.03  0.07  0.18  0.00  0.02  0.00  0.00  0.06  0.00  0.00  0.00  0.00  0.02  0.49  0.00 |  | 0.20 |  | 0.11 | 0.03 |  |  |  |  |  |  |
| AXIN2 | 0.00 | * | 0.44 |  | 0.04 | 0.03 |  |  |  |  |  |  |
| CCND1 | 0.00 | * | 0.01 | * | 0.09 | 0.03 | 0.75 | 0.01 | 0.03 | 0.09 | 0.04 | 0.03 |
| CDH1 | 0.03 | * | 0.14 |  | 0.06 | 0.03 |  |  |  |  |  |  |
| CDK2 | 0.07 |  | 0.45 |  | 0.02 | 0.02 |  |  |  |  |  |  |
| CDK4 | 0.18 |  | 0.35 |  | 0.02 | 0.02 |  |  |  |  |  |  |
| CDK6 | 0.00 | * | 0.50 |  | 0.03 | 0.02 |  |  |  |  |  |  |
| CDKN1A | 0.02 | * | 0.17 |  | 0.05 | 0.03 |  |  |  |  |  |  |
| E2F1 | 0.00 | * | 0.31 |  | 0.05 | 0.02 |  |  |  |  |  |  |
| EPHB2 | 0.00 | * | 0.74 |  | 0.01 | 0.02 |  |  |  |  |  |  |
| FOXM1 | 0.06 |  | 0.88 |  | -0.01 | 0.03 |  |  |  |  |  |  |
| ID1 | 0.00 | * | 0.53 |  | 0.04 | 0.03 |  |  |  |  |  |  |
| ID2 | 0.00 | * | 0.86 |  | -0.01 | 0.02 |  |  |  |  |  |  |
| ID3 | 0.00 | * | 0.76 |  | 0.03 | 0.04 |  |  |  |  |  |  |
| KCNH2 | 0.00 | * | 0.36 |  | 0.05 | 0.03 |  |  |  |  |  |  |
| KCNMA1 | 0.02 | * | 0.72 |  | 0.02 | 0.03 |  |  |  |  |  |  |
| LGR5 | 0.49 |  | 0.85 |  | -0.01 | 0.05 |  |  |  |  |  |  |
| LRIG1 | 0.00 | * | 0.49 |  | 0.02 | 0.02 |  |  |  |  |  |  |
| MCM2 | 0.00 | * | 0.87 |  | -0.01 | 0.02 |  |  |  |  |  |  |
| MKi67 | 0.20 |  | 0.32 |  | -0.03 | 0.02 |  |  |  |  |  |  |
| OLFM4 | 0.00 | * | 0.69 |  | -0.04 | 0.06 |  |  |  |  |  |  |
| PCBP4 | 0.00 | * | 0.01 | * | 0.10 | 0.02 | 0.69 | 0.02 | 0.05 | 0.09 | 0.05 | 0.03 |
| PLK1 | 0.00 | * | 0.77 |  | -0.02 | 0.03 |  |  |  |  |  |  |
| PMEPA1 | 0.45 |  | 0.00 | * | 0.16 | 0.03 | 0.95 | -0.00 | 0.05 | 0.10 | 0.06 | 0.04 |
| PRKCA | 0.45 |  | 0.56 |  | 0.02 | 0.02 |  |  |  |  |  |  |
| SCNN1A | 0.24 |  | 0.13 |  | 0.04 | 0.02 |  |  |  |  |  |  |
| SCNN1B | 0.00 | * | 0.32 |  | 0.05 | 0.04 |  |  |  |  |  |  |
| SCNN1D | 0.00 | * | 0.48 |  | 0.04 | 0.03 |  |  |  |  |  |  |
| SCNN1G | 0.00 | * | 0.96 |  | 0.01 | 0.08 |  |  |  |  |  |  |
| SNAI1 | 0.00 | * | 0.22 |  | 0.06 | 0.04 |  |  |  |  |  |  |
| SNAI2 | 0.00 | * | 0.15 |  | -0.11 | 0.05 |  |  |  |  |  |  |
| TFDP1 | 0.00 | * | 0.44 |  | 0.03 | 0.02 |  |  |  |  |  |  |
| TFDP2 | 0.06 |  | 0.33 |  | 0.06 | 0.02 |  |  |  |  |  |  |

**(b) Gene expression association with case/control status (proximal colon)**

| Gene | _p_phase1 | β_phase1 | SE_phase1 | p_phase2 |  | β_phase2 | SE_phase2 | p_phase1+2 |  | β phase1+2 | SE_phase1+2 |
| --- | --- | --- | --- | --- | --- | --- | --- | --- | --- | --- | --- |
| AURKA | 0.69 | 0.26 | 0.64 |  |  |  |  |  |  |  |  |
| AXIN2 | 0.86 | -0.14 | 0.82 |  |  |  |  |  |  |  |  |
| CCND1 | 0.51 | 1.01 | 1.54 | 0.79 |  | 0.26 | 0.98 | 0.66 |  | 0.36 | 0.83 |
| CDH1 | 0.98 | 0.03 | 1.03 |  |  |  |  |  |  |  |  |
| CDK2 | 0.65 | -0.79 | 1.74 |  |  |  |  |  |  |  |  |
| CDK4 | 0.83 | 0.42 | 1.98 |  |  |  |  |  |  |  |  |
| CDK6 | 0.60 | -0.62 | 1.19 |  |  |  |  |  |  |  |  |
| CDKN1A | 0.48 | -0.88 | 1.25 |  |  |  |  |  |  |  |  |
| E2F1 | 0.95 | -0.07 | 1.15 |  |  |  |  |  |  |  |  |
| EPHB2 | 0.80 | 0.37 | 1.51 |  |  |  |  |  |  |  |  |
| FOXM1 | 0.45 | -1.22 | 1.62 |  |  |  |  |  |  |  |  |
| ID1 | 0.81 | 0.18 | 0.75 |  |  |  |  |  |  |  |  |
| ID2 | 0.67 | 0.74 | 1.75 |  |  |  |  |  |  |  |  |
| ID3 | 0.71 | -0.18 | 0.49 |  |  |  |  |  |  |  |  |
| KCNH2 | 0.66 | -0.35 | 0.79 |  |  |  |  |  |  |  |  |
| KCNMA1 | 0.48 | -0.72 | 1.04 |  |  |  |  |  |  |  |  |
| LGR5 | 0.43 | -0.52 | 0.66 |  |  |  |  |  |  |  |  |
| LRIG1 | 0.79 | -0.39 | 1.45 |  |  |  |  |  |  |  |  |
| MCM2 | 0.85 | 0.31 | 1.59 |  |  |  |  |  |  |  |  |
| MKI67 | 0.81 | -0.28 | 1.21 |  |  |  |  |  |  |  |  |
| OLFM4 | 0.75 | 0.29 | 0.94 |  |  |  |  |  |  |  |  |
| PCBP4 | 0.88 | -0.22 | 1.39 | 0.03 | * | 1.77 | 0.82 | 0.07 |  | 1.24 | 0.68 |
| PLK1 | 1.00 | 0.00 | 0.94 |  |  |  |  |  |  |  |  |
| PMEPA1 | 0.15 | 1.57 | 1.08 | 0.17 |  | 0.84 | 0.62 | 0.03 | * | 1.23 | 0.56 |
| PRKCA | 0.84 | 0.33 | 1.62 |  |  |  |  |  |  |  |  |
| SCNN1A | 0.95 | 0.09 | 1.52 |  |  |  |  |  |  |  |  |
| SCNN1B | 0.72 | 0.33 | 0.94 |  |  |  |  |  |  |  |  |
| SCNN1D | 0.54 | -0.57 | 0.93 |  |  |  |  |  |  |  |  |
| SCNN1G | 0.63 | 0.20 | 0.40 |  |  |  |  |  |  |  |  |
| SNAI1 | 0.34 | -1.03 | 1.07 |  |  |  |  |  |  |  |  |
| SNAI2 | 0.26 | -0.84 | 0.74 |  |  |  |  |  |  |  |  |
| TFDP1 | 0.66 | -0.48 | 1.07 |  |  |  |  |  |  |  |  |
| TFDP2 | 0.89 | 0.10 | 0.74 |  |  |  |  |  |  |  |  |

**(c) Gene expression association with case/control status (distal colon)**

| Gene | p_phase1 |  | β_phase1 | SE_phase1 | p_phase2 |  | β_phase2 | SE_phase2 | p_phase1+2 |  | β phase1+2 | SE_phase1+2 |
| --- | --- | --- | --- | --- | --- | --- | --- | --- | --- | --- | --- | --- |
| AURKA | 0.39 |  | 0.91 | 1.06 |  |  |  |  |  |  |  |  |
| AXIN2 | 0.58 |  | 0.63 | 1.13 |  |  |  |  |  |  |  |  |
| CCND1 | 0.22 |  | 1.56 | 1.27 | 0.39 |  | 0.79 | 0.93 | 0.16 |  | 1.02 | 0.74 |
| CDH1 | 0.14 |  | 2.24 | 1.52 |  |  |  |  |  |  |  |  |
| CDK2 | 0.63 |  | 0.84 | 1.72 |  |  |  |  |  |  |  |  |
| CDK4 | 0.74 |  | 0.67 | 2.06 |  |  |  |  |  |  |  |  |
| CDK6 | 0.81 |  | -0.28 | 1.17 |  |  |  |  |  |  |  |  |
| CDKN1A | 0.14 |  | 1.87 | 1.26 |  |  |  |  |  |  |  |  |
| E2F1 | 0.36 |  | 1.10 | 1.20 |  |  |  |  |  |  |  |  |
| EPHB2 | 0.44 |  | -1.05 | 1.35 |  |  |  |  |  |  |  |  |
| FOXM1 | 0.23 |  | -1.47 | 1.22 |  |  |  |  |  |  |  |  |
| ID1 | 0.11 |  | 1.94 | 1.21 |  |  |  |  |  |  |  |  |
| ID2 | 0.29 |  | 1.94 | 1.82 |  |  |  |  |  |  |  |  |
| ID3 | 0.24 |  | 1.00 | 0.84 |  |  |  |  |  |  |  |  |
| KCNH2 | 0.34 |  | 0.78 | 0.81 |  |  |  |  |  |  |  |  |
| KCNMA1 | 0.10 |  | 2.13 | 1.31 |  |  |  |  |  |  |  |  |
| LGR5 | 0.11 |  | -1.75 | 1.11 |  |  |  |  |  |  |  |  |
| LRIG1 | 0.86 |  | -0.21 | 1.16 |  |  |  |  |  |  |  |  |
| MCM2 | 0.45 |  | -1.19 | 1.56 |  |  |  |  |  |  |  |  |
| MKI67 | 0.27 |  | -1.45 | 1.33 |  |  |  |  |  |  |  |  |
| OLFM4 | 0.55 |  | -0.28 | 0.46 |  |  |  |  |  |  |  |  |
| PCBP4 | 0.07 |  | 2.60 | 1.41 | 0.89 |  | 0.11 | 0.81 | 0.20 |  | 0.88 | 0.69 |
| PLK1 | 0.57 |  | -0.54 | 0.95 |  |  |  |  |  |  |  |  |
| PMEPA1 | 0.02 | * | 2.17 | 0.92 | 0.85 |  | -0.010 | 0.54 | 0.13 |  | 0.68 | 0.45 |
| PRKCA | 0.27 |  | 1.71 | 1.55 |  |  |  |  |  |  |  |  |
| SCNN1A | 0.03 | * | 3.77 | 1.71 | 0.01 | * | 1.21 | 0.49 | 0.00 | * | 1.41 | 0.49 |
| SCNN1B | 0.07 |  | 1.65 | 0.91 |  |  |  |  |  |  |  |  |
| SCNN1D | 0.59 |  | 0.40 | 0.74 |  |  |  |  |  |  |  |  |
| SCNN1G | 0.75 |  | 0.16 | 0.50 |  |  |  |  |  |  |  |  |
| SNAI1 | 0.11 |  | 1.29 | 0.82 |  |  |  |  |  |  |  |  |
| SNAI2 | 0.76 |  | 0.17 | 0.56 |  |  |  |  |  |  |  |  |
| TFDP1 | 0.88 |  | 0.16 | 1.07 |  |  |  |  |  |  |  |  |
| TFDP2 | 0.52 |  | 0.56 | 0.88 |  |  |  |  |  |  |  |  |

**(d) Gene expression association with case/control status (rectum)**

| Gene | p_phase1 |  | β_phase1 | SE_phase1 | p phase2 | β phase2 | SE_phase2 | p_phase1+2 | β phase1+2 | SE_phase1+2 |
| --- | --- | --- | --- | --- | --- | --- | --- | --- | --- | --- |
| AURKA | 0.23 |  | 1.96 | 1.63 |  |  |  |  |  |  |
| AXIN2 | 0.86 |  | -0.32 | 1.82 |  |  |  |  |  |  |
| CCND1 | 0.54 |  | 1.27 | 2.06 | 0.58 | 0.46 | 0.82 | 0.34 | 0.80 | 0.83 |
| CDH1 | 0.33 |  | -2.46 | 2.52 |  |  |  |  |  |  |
| CDK2 | 0.33 |  | 2.06 | 2.09 |  |  |  |  |  |  |
| CDK4 | 0.32 |  | 2.59 | 2.58 |  |  |  |  |  |  |
| CDK6 | 0.42 |  | 1.61 | 1.99 |  |  |  |  |  |  |
| CDKN1A | 0.36 |  | -2.08 | 2.25 |  |  |  |  |  |  |
| E2F1 | 0.04 | * | 3.14 | 1.55 | 0.56 | 0.06 | 0.11 | 0.45 | 0.08 | 0.11 |
| EPHB2 | 0.78 |  | -0.58 | 2.12 |  |  |  |  |  |  |
| FOXM1 | 0.95 |  | 0.12 | 1.92 |  |  |  |  |  |  |
| ID1 | 0.99 |  | -0.02 | 1.53 |  |  |  |  |  |  |
| ID2 | 0.56 |  | -1.27 | 2.17 |  |  |  |  |  |  |
| ID3 | 0.46 |  | 0.82 | 1.11 |  |  |  |  |  |  |
| KCNH2 | 0.04 | * | 4.55 | 2.21 | 0.18 | -0.82 | 0.61 | 0.60 | -0.27 | 0.51 |
| KCNMA1 | 0.57 |  | -0.99 | 1.75 |  |  |  |  |  |  |
| LGR5 | 0.17 |  | 1.54 | 1.13 |  |  |  |  |  |  |
| LRIG1 | 0.05 |  | 4.91 | 2.49 |  |  |  |  |  |  |
| MCM2 | 0.78 |  | -0.65 | 2.35 |  |  |  |  |  |  |
| MKI67 | 0.81 |  | -0.41 | 1.74 |  |  |  |  |  |  |
| OLFM4 | 0.90 |  | -0.08 | 0.62 |  |  |  |  |  |  |
| PCBP4 | 0.02 | * | 5.01 | 2.08 | 0.13 | -1.21 | 0.80 | 0.97 | 0.02 | 0.62 |
| PLK1 | 0.95 |  | -0.08 | 1.14 |  |  |  |  |  |  |
| PMEPA1 | 0.27 |  | 1.37 | 1.23 | 0.26 | -0.76 | 0.67 | 0.74 | -0.20 | 0.59 |
| PRKCA | 0.54 |  | 1.27 | 2.05 |  |  |  |  |  |  |
| SCNN1A | 0.75 |  | 0.71 | 2.27 |  |  |  |  |  |  |
| SCNN1B | 0.81 |  | -0.38 | 1.53 |  |  |  |  |  |  |
| SCNN1D | 0.38 |  | 1.09 | 1.25 |  |  |  |  |  |  |
| SCNN1G | 0.99 |  | -0.01 | 0.72 |  |  |  |  |  |  |
| SNAI1 | 0.58 |  | 0.67 | 1.21 |  |  |  |  |  |  |
| SNAI2 | 0.88 |  | -0.15 | 0.96 |  |  |  |  |  |  |
| TFDP1 | 0.21 |  | 3.70 | 2.93 |  |  |  |  |  |  |
| TFDP2 | 0.28 |  | 2.08 | 1.92 |  |  |  |  |  |  |

**Supplementary Table 4. Association results for pathway expression by case/control status**. We show β, SE, p-value and FDR-corrected q-value for the “all bowel” test and sub-analyses by location (proximal colon, distal colon and rectum). NA=not available owing to number of data points being below minimum for PCA analysis.

| Pathway | β all bowel | SE all bowel | P all bowel | q all bowel | β proximal | SE proximal | p  proximal | q  proximal |
| --- | --- | --- | --- | --- | --- | --- | --- | --- |
| Stem cell | -0.360 | 0.339 | 0.288 | 0.576 | 2.665 | 0.554 | 1.529E-06 | 1.223E-05* |
| p21 | 0.928 | 0.711 | 0.192 | 0.576 | -0.508 | 0.376 | 0.176 | 0.235 |
| Wnt | 0.644 | 0.576 | 0.263 | 0.576 | -1.250 | 0.519 | 0.016 | 0.040* |
| BMP | 0.305 | 0.455 | 0.503 | 0.805 | 0.568 | 0.636 | 0.372 | 0.425 |
| Proliferation | 0.088 | 0.340 | 0.795 | 0.849 | 0.428 | 0.792 | 0.589 | 0.589 |
| Colonocyte differentiation | 1.631 | 0.636 | 0.010 | 0.080 | 2.513 | 1.078 | 0.020 | 0.040* |
| Ion channels | 0.075 | 0.328 | 0.819 | 0.849 | 1.267 | 0.372 | 0.001 | 0.004* |
| EMT | -0.075 | 0.395 | 0.849 | 0.849 | -0.848 | 0.471 | 0.072 | 0.115 |

| Pathway | β  distal | SE  distal | p  distal | q  distal | β  rectum | SE rectum | p  rectum | q  rectum |
| --- | --- | --- | --- | --- | --- | --- | --- | --- |
| Stem cell | -1.121 | 0.394 | 0.004 | 0.032* | -0.504 | 0.523 | 0.335 | 0.402 |
| p21 | 0.494 | 1.038 | 0.634 | 0.845 | NA | NA | NA | NA |
| Wnt | 0.249 | 0.874 | 0.775 | 0.847 | NA | NA | NA | NA |
| BMP | 1.183 | 0.777 | 0.128 | 0.341 | 0.670 | 0.573 | 0.243 | 0.365 |
| Proliferation | -0.329 | 0.470 | 0.484 | 0.774 | 0.847 | 1.089 | 0.437 | 0.437 |
| Colonocyte differentiation | 1.745 | 0.821 | 0.034 | 0.136 | 2.528 | 1.139 | 0.026 | 0.078 |
| Ion channels | -0.327 | 0.339 | 0.335 | 0.670 | -1.603 | 0.444 | 3.064E-04 | 0.002* |
| EMT | -0.127 | 0.659 | 0.847 | 0.847 | 1.079 | 0.794 | 0.174 | 0.348 |

**Supplementary Figure 1.** **Telomere length by location in the large bowel.** Box plot of relative telomere length values for the proximal and distal colon, and rectum with the corresponding paired Wilcoxon test p values.

**Supplementary Figure 2. Scatter plot showing the correlation between bowel and blood global TL.** Orange line shows the predicted linear fit.

**Supplementary Figure 3. Association of 5mC levels with case/control status.** Beeswarm plot of 5mC values with overlying boxplot.

**Supplementary Figure 4. Scatter plot showing the correlation between bowel and blood 5mC levels.** The orange line shows the predicted linear fit.

**Supplementary Figure 5. Methylation by location in the bowel.** Box plot of global methylation (5mC) values for the proximal and distal colon and rectum, showing lower methylation levels in the proximal colon. p values from the corresponding paired Wilcoxon test are also shown.

**Supplementary Figure 6. Differences in gene expression among the three regions of the large bowel.** We show for each pairwise comparison between sites of the bowel (proximal vs distal colon, distal colon vs rectum and proximal colon vs rectum) the genes that have significant differential expression. *AURKA, CDK4, ID1, LGR5, MKI67, TFDP1 and TFDP2* showed consistent expression levels throughout the bowel and hence do not appear in the figure.

**Supplementary Figure 7. Box plots of expression levels of each gene by bowel location.** Box colour corresponds to pathway as per Supplementary Figure 1.

**Supplementary Figure 8. Pairwise correlation heatmap between gene expression levels.** Plot shows the correlation coefficients for all pairwise combinations of genes. Highlighted zones correspond to correlation values for genes in the same pathway (green=positive, purple=negative, darker colour=stronger association). Gene colour depicts pathway (black=stem cell markers; red=senescence; blue=Wnt; yellow=BMP; dark green=proliferation; green=colonocyte differentiation; pink=colonocyte ion channels; purple=EMT).

**Supplementary Figure 9. Expression by case/control status of *SCNN1A* in the distal colon.** Fold changes in expression relative to the median of the control group are shown.

**Supplementary Figure 10. Association of polygenic risk score with case/control status.** Beeswarm with overlying boxplot for polygenic risk score. Other annotation is as per Figure 1.

**Supplementary Figure 11. Relationship between bowel telomere length and telomerase complex SNP genotype.** Boxplot showing the distribution of TL per genotype of: A) rs2735940; B) rs10936599 and C) rs2736100. For rs2735940 and rs10936599 we can observe the significant increase in TL per risk allele.
